# Anti-Inflammatory Effects and Mechanisms of *Fatsia polycarpa* Hayata and Its Constituents

**DOI:** 10.1155/2013/857213

**Published:** 2013-12-10

**Authors:** Hsueh-Ling Cheng, Nurkholis, Shi-Yie Cheng, Shen-Da Huang, Yan-Ting Lu, Xiao-Wen Wang, Yu-Liang Liu, Chang-Hung Chou

**Affiliations:** ^1^Department of Biological Science and Technology, National Pingtung University of Science and Technology, No. 1, Shuefu Road, Neipu, Pingtung 91201, Taiwan; ^2^Department of Agricultural Product Technology, Brawijaya University, Jalan, Veteran Malang 65145, Indonesia; ^3^Department of Life Sciences, National University of Kaohsiung, No. 700, Kaohsiung University Road, Nan-Tzu District, Kaohsiung 81148, Taiwan; ^4^Research Center for Biodiversity and Graduate Institute of Ecology and Evolutionary Biology, China Medical University, 91 Hsueh-Shih Road, Taichung 40402, Taiwan

## Abstract

*Fatsia polycarpa*, a plant endemic to Taiwan, is an herbal medicine known for treating several inflammation-related diseases, but its biological function needs scientific support. Thus, the anti-inflammatory effects and mechanisms of the methanolic crude extract (MCE) of *F. polycarpa* and its feature constituents, that is, brassicasterol (a phytosterol), triterpenoids 3**α**-hydroxyolean-11,13(18)-dien-28-oic acid (HODA), 3**α**-hydroxyolean-11-en-28,13**β**-olide (HOEO), fatsicarpain D, and fatsicarpain F, were investigated. MCE and HOEO, but not brassicasterol, dose-dependently inhibited lipopolysaccharide- (LPS-)induced expression of inducible nitric oxide synthase and cyclooxygenase-2 in RAW 264.7 macrophage line, whereas HODA, fatsicarpain D and fatsicarpain F were toxic to RAW cells. Additionally, MCE and HOEO suppressed LPS-induced production of nitric oxide, prostaglandin E_2_, and interleukin-1**β** and interfered with LPS-promoted activation of the inhibitor kappa B kinase (IKK)/nuclear factor-**κ**B (NF-**κ**B) pathway, and that of the mitogen-activated protein kinases (MAPKs) extracellular signal regulated kinase (ERK), c-Jun N-terminal kinase (JNK), and p38. In animal tests, MCE and HOEO effectively ameliorated 12-O-tetradecanoylphorobol-13 acetate- (TPA-)induced ear edema of mice. Thus, MCE of *F. polycarpa* exhibited an obvious anti-inflammatory activity *in vivo* and *in vitro* that likely involved the inhibition of the IKK/NF-**κ**B pathway and the MAPKs, which may be attributed by triterpenoids such as HOEO.

## 1. Introduction

There are three species in the genus of *Fatsia* (belonging to the Araliaceae family), that is, *Fatsia polycarpa* Hayata, an evergreen shrub endemic to Taiwan, *Fatsia japonica,* and *Fatsia oligocarpela*, originated from Japan and Bonin Islands, respectively [1]. *F. japonica* and *F. polycarpa* have been used as an herbal medicine in Japan and in Taiwan in treating diseases, such as coughing, ankylosing spondylitis, osteoarthritis, rheumatism, rheumatoid arthritis and tendinitis, and in blood circulation improvement [1, 2]. Nonetheless, the therapeutic activity of *F. polycarpa* has seldom been scientifically investigated and the bioactive constituents from these plants are not clear.

The constituents in the methanolic extract of leaves and twigs of *Fatsia polycarpa* were previously characterized [1]. The extract was rich in phytosterols in which brassicasterol was a major one (unpublished data). Brassicasterol is a common phytosterol present in many plants and was suggested to reduce blood cholesterol levels together with other phytosterols [3]. Triterpenoids is another type of compounds rich in the crude extract of *F. polycarpa* [1, 4, 5]. Seven novel structures (named fatsicarpains A–G) and two known ones (3**α**-hydroxyolean-11,13(18)-dien-28-oic acid (HODA) and 3**α**-hydroxyolean-11-en-28,13**β**-olide (HOEO)) belonging to oleanane-type triterpenoids were isolated and identified from the extract [1]. Among these triterpenes, HODA, HOEO, fatsicarpain D, and fatsicarpain F are in larger amounts. Whether these molecules play roles in the medical functions of* Fatsia polycarpa* deserves to be investigated.

**Figure 1:**
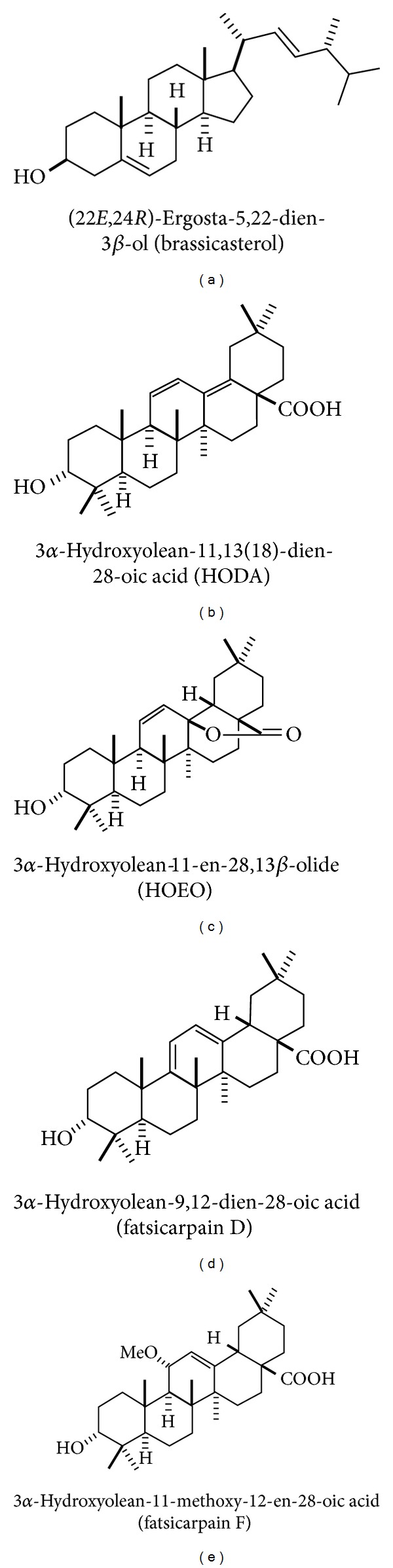
Structures of brassicasterol (a), HODA (b), HOEO (c), fatsicarpain D (d), and fatsicarpain F (e).

*Fatsia* plants exhibit pleiotropic therapeutic activities as described above. However, several of these activities, such as the treatment of ankylosing spondylitis, osteoarthritis, rheumatoid arthritis, and tendinitis, are related to anti-inflammation. Thus, in this study, the anti-inflammatory effects of the methanolic crude extract (MCE) of *F. polycarpa* and its major or feature constituents, including brassicasterol, HODA, HOEO, fatsicarpain D and fatsicarpain F (structures shown in Figure 1), were characterized.

## 2. Materials and Methods

### 2.1. Chemicals

Specific antibodies against phosphorylated IKK-**α**/IKK-**β**, total IKK-**α**, total IKK-**β**, phosphorylated I*κ*B-**α**, total I*κ*B-**α**, phosphorylated JNK, total JNK, phosphorylated ERK, total ERK, phosphorylated p38, total p38, p65, and lamin B were purchased from Cell Signaling Technology (Beverly, MA, USA); antibodies against inducible nitric oxide synthase and cyclooxygenase-2 from BD Biosciences (Franklin Lakes, CA, USA); an actin-specific antibody from Chemicon (Temecula, CA, USA); all secondary antibodies from Santa Cruz Biotechnologies (Santa Cruz, CA, USA); fetal bovine serum (FBS) from Invitrogen (San Diego, CA, USA); and cell culture media, lipopolysaccharide (LPS) derived from *E. coli 055:B5*, NaF, sodium orthovanadate, sodium pyrophosphate, indomethacin, MTT (3-(4,5-dimethylthiazol-2-yl)-2,5-diphenyltetrazolium bromide), and 12-O-tetradecanoylphorobol-13 acetate (TPA) from Sigma-Aldrich (St. Louis, MO, USA) at reagent grade or cell culture grade.

### 2.2. Isolation and Identification of MCE, Brassicasterol, HODA, HOEO, Fatsicarpain D, and Fatsicarpain F

The air-dried and powdered leaves and twigs of *F. polycarpa* (7.1 kg) were extracted with methanol (MeOH) for three days at room temperature (three times) and the combined extracts were concentrated in vacuum (under 35°C). The resulting dark green gum, that is, MCE, was suspended in H_2_O and extracted sequentially with CH_2_Cl_2_, ethyl acetate (EtOAc), and *n*-butanol (*n*-BuOH) (saturated with H_2_O). The CH_2_Cl_2_ extract (100 g) was subjected to column chromatography on silica gel using *n*-hexane, *n*-hexane-EtOAc, and EtOAc-MeOH mixtures of increasing polarity for elution to furnish 40 fractions. Fraction 20 (7.0 g) eluted with *n*-hexane-EtOAc (1 : 10) was fractionated over Sephadex LH-20 (100% acetone) to afford a major phytosterol (580 mg), which was grown by slow evaporation of the mixture CH_2_Cl_2_-MeOH (1 : 1) solution at room temperature to give a suitable colorless crystal. The chemical structure of the crystal was confirmed by comparison of its spectroscopic data with those of brassicasterol [6, 7], which was reported specifically in only one alga, an unidentified species of the order Sarcinochrysidales (Chrysophyceae).

HODA, HOEO, fatsicarpain D, and fatsicarpain F were prepared, or isolated and identified as described previously [1]. Briefly, fraction 17 (2.86 g) of the CH_2_Cl_2_ extract (100 g), eluted with *n-*hexane-EtOAc (1 : 4), was fractionated over Sephadex LH-20 (100% acetone) to afford HODA (108 mg). Fraction 20 (6.97 g), eluted with *n*-hexane-EtOAc (1 : 10), was chromatographed over silica gel, eluted in a step gradient manner with *n*-hexane-EtOAc-MeOH (10 : 1 : 0 to 0 : 0 : 100) to afford six subfractions. Subfraction 20–2 (3.16 g) was fractionated over Sephadex LH-20 (100% acetone) to yield a mixture (775 mg) that was subjected to RP-18 column chromatography eluting with 65% MeOH in H_2_O, 75% MeOH in H_2_O, 85% MeOH in H_2_O, 90% MeOH in H_2_O, and 100% MeOH. Five subfractions were obtained, of which subfraction 4 (258 mg) was submitted to repeated column chromatography over RP-18 using MeOH-H_2_O-MeCN (20 : 60 : 20 + 0.2% FA (formic acid)) to give a mixture (74 mg) that was further separated by RP-18 HPLC using H_2_O-MeCN (35 : 65 + 0.3% FA) to isolate fatsicarpain D (4 mg) and HOEO (40 mg). Fraction 24 (1.5 g), eluted with EtOAc-MeOH (70 : 1), was subjected to silica gel column chromatography (*n*-hexane-EtOAc-MeOH, from 10 : 1 : 0 to 0 : 10 : 1) to obtain 10 subfractions. Subfraction 24–6 (306 mg), eluted with *n*-hexane-EtOAc (1 : 3), was subsequently fractionated over Sephadex LH-20 (100% acetone) to yield a mixture that was further purified by RP-18 HPLC using MeOH-H_2_O-MeCN (70 : 25 : 5) to give fatsicarpain F (2 mg).

MCE was dissolved in DMSO (dimethylsulfoxide) in a concentration of 50 mg/mL as a stock solution. Brassicasterol was dissolved in 100% ethanol in a concentration of 10 mM as a stock solution. HODA, HOEO, fatsicarpain D, and fatsicarpain F were dissolved in DMSO in a concentration of 10 mM as the stock solutions.

### 2.3. Animal Tests

All protocols of animal tests were approved by the Committee for Animal Experiments of National Pingtung University of Science and Technology in accordance with international guidelines. Experiments were performed using groups of 8 male ICR mice (30–35 g; purchased from BioLASCO, Taipei, Taiwan) fed with a regular laboratory rodent diet and housed under a 12-hour light-dark cycle. TPA-induced ear edema of mice was performed according to previously published methods with modifications [8, 9]. In the experiment for MCE, TPA (2.5 *μ*g/ear dissolved in 20 *μ*L of acetone) was topically applied to the surfaces of both ears of mice, whereas in the group of TPA control, TPA was applied to only the right ear. Meanwhile, in the group of normal control, 20 *μ*L of acetone was applied to both ears. Four hours later, MCE (100, 300, or 500 *μ*g/ear) or indomethacin (500 *μ*g/ear), both dissolved in acetone, was applied to the surface of the right ear, and acetone to the left ear, whereas for normal control and TPA control, acetone was applied to both ears. The thickness of ears was measured before and at 4 h, 16 h, and 24 h after TPA treatment using a dial thickness gauge (Peacock, Ozaki, Tokyo, Japan). The experiment for HOEO was performed similarly using a different batch of mice, but each group contained 6 mice, the dosage of TPA was 3.0 *μ*g/ear, and HOEO (300 *μ*g/ear) or indomethacin (500 *μ*g/ear) was applied to the ears at 1 h (instead of 4 h) after TPA treatment.

### 2.4. Cell Culture and Western Blot Analysis

RAW 264.7 cells (ATCC number AT TIB-71) were cultured in DMEM (Dulbecco's modified Eagle's medium) containing 10% fetal bovine serum. FL83B cells (ATCC number CRL-2390) were cultured as previously described [10]. Both cells were incubated at 37°C in a humidified incubator supplied with 5% CO_2_.

RAW 264.7 cells were seeded at a density of 1.5−2.0 × 10^6^/35-mm plate, grown for 16 h, and treated with MCE or the natural products for 3 h. LPS was then added to the medium (final concentration 100 ng/mL) for 16 h. Subsequently, cells were washed with cold PBS (phosphate-buffered saline, pH 7.4) twice and submerged in lysis buffer (1X Cell Culture Lysis Reagent (Promega, Madison, WI, USA) containing 1 mM of phenylmethylsulfonyl fluoride, 1 *μ*g/mL of pepstatin, 1 *μ*g/mL of leupeptin, and 1 *μ*g/mL of aprotinin). For the detection of phosphorylated proteins, the lysis buffer also contained 10 mM of NaF, 1 mM of sodium orthovanadate, and 10 mM of sodium pyrophosphate. Cells were scraped off the plate on ice and the suspension was centrifuged at 14,000 ×g for 15 min at 4°C. The supernatant was collected and the protein concentration was analyzed using Bradford assay reagent (Bio-Rad, Hercules, CA, USA). Equal amounts of proteins were sampled and subjected to electrophoresis and Western blotting as described previously [11]. The resulting blots were scanned by a gel documentation and image analysis system (Syngene, Frederick, MD, USA), and band intensities were analyzed using the program supplied with the system. Alternatively, after hybridization with a horseradish-peroxidase- (HRP-) conjugated secondary antibody, followed by incubation with “Western HRP substrate” (Luminata Classico, Millipore, Temecula, CA, USA), immunoreactive bands were detected using a biospectrum imaging system (UVP Biospectrum, UVP, LLC, Upland, CA, USA) and band intensities were analyzed by the supplied software.

### 2.5. Cytotoxicity Assay

Cytotoxicity was assayed using MTT. RAW 264.7 (3 × 10^5^ cell/well) or FL83B cells (2 × 10^5^ cell/well) were cultured in a 96-well plate. Cells were washed twice with PBS, treated with the compounds in the indicated concentrations in serum-free medium for 24 h (HODA) or 19 h (other compounds). Cells were then washed twice with PBS and incubated in 30 *μ*L of 5 mg/mL MTT solution at 37°C in the dark for 1 h. Subsequently, DMSO (100 *μ*L) was added to each well for 10 minutes, withdrawn to another 96-well plate, and the absorbance at 570 nm determined using a microplate reader (Molecular Devices, Sunnyvale, CA, USA). Experiments were performed in triplicate. Average ratio ± standard deviation of viable cells was calculated against the control (cells treated by the solvent).

### 2.6. Semiquantitative RT-PCR (Reverse Transcriptase-Polymerase Chain Reaction)

Total cellular RNA was purified using Trizol (Invitrogen) as per the manufacturer's instruction. The quality of RNA was checked by agarose gel electrophoresis. Sequences of the forward and reverse PCR primers of interleukin-1**β** (IL-1**β**) and glyceraldehyde-3-phosphate dehydrogenase (GAPDH) were as published previously [12]. RT-PCR was performed using the SuperScript One-Step RT-PCR System kit (Invitrogen) with the following formulation: 2 *μ*g of total RNA, 0.4 *μ*M of forward primer and reverse primer, respectively, 12.5 *μ*L of 2X reaction mix, and 1 *μ*L of SuperScript RT/Platinum *Taq*. The final volume was adjusted to 25 *μ*L with sterilized distilled water. It was then subjected to PCR amplification in a thermal cycler by the following program: 48°C, 45 min (for cDNA synthesis); one cycle of 95°C, 2 min, 35 cycles of 95°C, 45 s, 60°C, 45 s, and 72°C, 1 min, and one cycle of 72°C, 5 min. The RT-PCR products were separated electrophoretically on 1% agarose gels and visualized by ethidium bromide staining. The resulting images were recorded and band intensities were quantified as described previously [12]. Meanwhile, the RT-PCR products were purified and the sequences confirmed by DNA sequencing (Mission Biotech, Taipei, Taiwan).

### 2.7. Analysis of Nitric Oxide (NO) and Prostaglandin E_**2**_ (PGE_**2**_)

The concentration of NO in the culture medium of cells was analyzed using a kit of Griess reagent according to the manufacturer's protocol (Promega). That of PGE_2_ was analyzed using an ELISA kit as per the protocol suggested by the manufacturer (R&D Systems, Minneapolis, MN, USA).

### 2.8. Protein Extraction from the Nuclear and Cytoplasmic Fractions of Cells

RAW 264.7 cells were seeded on 10 mm dishes until 80% confluence and treated with MCE or HOEO for 3 h, followed by treating with LPS for 2 h. Cells were harvested, washed with cold PBS twice, and centrifuged at 500 ×g for 5 min. Nuclear proteins and cytoplasmic proteins of the cell pellet were then separated and extracted using a nuclear extract kit as per the manufacturer's instruction (Millipore, Temecula, CA, USA). Protein concentrations in the extracts were quantified using Bradford assay reagent before being subjected to Western blot analysis.

### 2.9. Statistical Analysis

Data were analyzed by one-way analysis of variance (ANOVA) followed by Scheffe's post hoc test and significance was considered when *P* < 0.05 and *F* > 3.5546.

## 3. Results

### 3.1. *In Vivo* Efficacy of MCE in TPA-Induced Mouse Ear Edema

**Figure 2:**
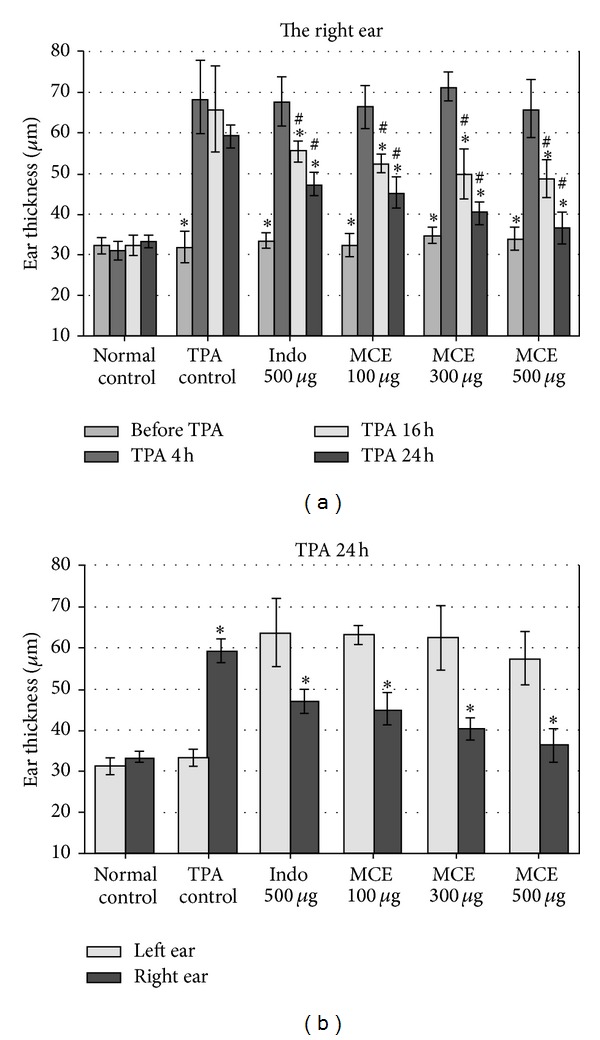
The anti-inflammatory effect of MCE on TPA-induced ear edema of mice. Mice were treated with TPA on both ears for 4 h, followed by MCE (100, 300, or 500 *μ*g/ear) or indomethacin (Indo; 500 *μ*g/ear) administration on the right ear, while the left ear was unmedicated. In TPA control, TPA was applied to only the right ear; in normal control, an equal amount of vehicle was applied to both ears. Ear thickness was measured before and at 4 h, 16 h, and 24 h after TPA treatment. Data represent the mean ± SD of each group (*N* = 8) and were statistically analyzed by one-way ANOVA. (a) The comparison of the thickness of the right ear at different time points. *P < 0.05 against “TPA 4 h” of the same group; ^#^ *P* < 0.05 versus the same time point in the group of TPA control. (b) The comparison of the thickness of the right ear and the left ear at 24 h after TPA treatment. *P < 0.05 against the left ear of the same group.

To assess the anti-inflammatory effect of MCE* in vivo*, a topical inflammation model, TPA-induced mouse ear edema, was used. Both ears of mice were stimulated with TPA to induce inflammation. The right ear was treated with MCE (100, 300, or 500 *μ*g/ear) or indomethacin (500 *μ*g/ear, as a reference drug) [13, 14] at 4 h after TPA stimulation, whereas the left ear remained unmedicated for comparison. As shown in Figure 2(a), ear edema was obvious at 4 h, 16 h, and 24 h after TPA application without any medication (TPA control), while all three dosages of MCE significantly reduced the edema of the right ear at 16 h and 24 h in a time-dependent manner and in an extent comparable to the effect of indomethacin. While comparing the thickness of the right ear and that of the unmedicated left ear at 24 h after TPA stimulation, the data also revealed that all three dosages of MCE effectively ameliorated the TPA-induced edema (Figure 2(b)). These results confirmed the anti-inflammatory activity of MCE *in vivo*. Accordingly, the anti-inflammatory effects of MCE and its characteristic components brassicasterol, HODA, HOEO, fatsicarpain D, and fatsicarpain F were further investigated.

### 3.2. The Anti-Inflammatory Effects of MCE and Its Feature Constituents on Macrophage Cells

**Figure 3:**
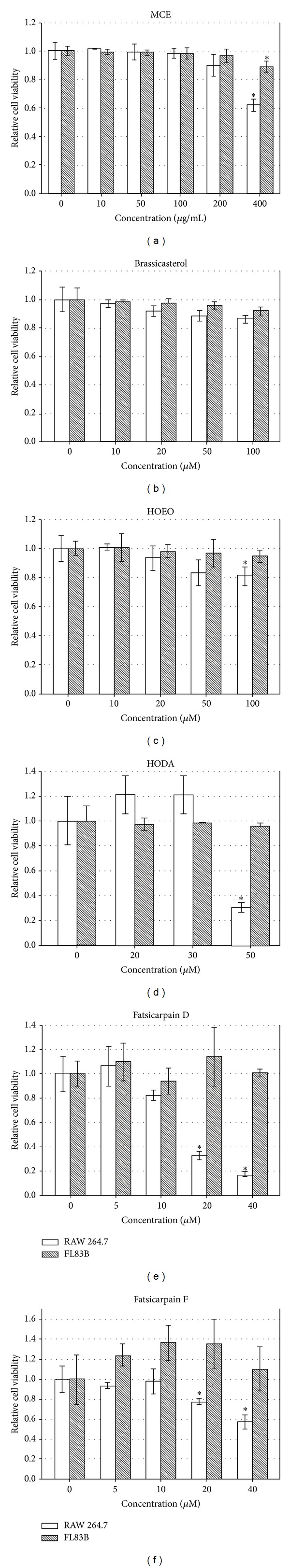
Cytotoxicity assays of MCE (a), brassicasterol (b), HOEO (c), HODA (d), fatsicarpain D (e), and fatsicarpain F (f). RAW 264.7 cells and FL83B cells were treated with the selected compound in the indicated concentrations for 24 h (HODA) or 19 h (other compounds). Relative cell viability was determined against the control (0 *μ*g/mL or 0 *μ*M of the compound). Data represent the mean ± SD of triplicate. *P < 0.05 against the control.

The cytotoxicity of MCE and the compounds was evaluated in RAW 264.7 macrophage cell line and in FL83B normal liver cell line. MCE did not display obvious cytotoxicity in FL83B cells in concentrations of 10–400 *μ*g/mL but did result in reduced cell survival in 400 *μ*g/mL in RAW 264.7 cells (Figure 3(a)). Brassicasterol did not show obvious cytotoxicity in both cell lines in 10–100 *μ*M (Figure 3(b)). HOEO did not have a significant toxic effect in FL83B cells in concentrations of 10–100 *μ*M but did result in about 20% inhibition for the growth of RAW 264.7 cells in 100 *μ*M (Figure 3(c)). HODA, fatsicarpain D, and fatsicarpain F on the other hand, obviously suppressed the growth of RAW 264.7 cells in concentrations of 50 *μ*M or ≧20 *μ*M, but they were not toxic to FL83B cells (Figures 3(d), 3(e), and 3(f), resp.). Therefore, the anti-inflammatory effects of MCE, brassicasterol, and HOEO were further characterized using RAW 264.7 cells as a model. Those of HODA, fatsicarpain D, and fatsicarpain F will be analyzed using other appropriate models.

**Figure 4:**
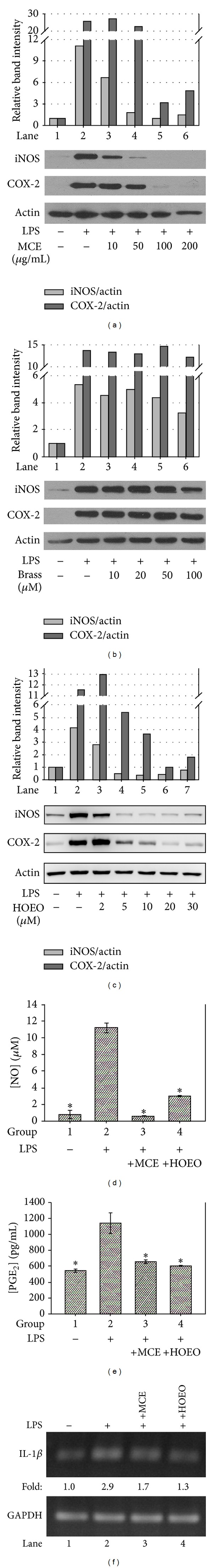
Suppression of the expression of LPS-induced proinflammatory factors by MCE or HOEO. (a), (b), and (c). The analysis by Western blot of the expression of iNOS and COX-2 in RAW 264.7 cells. Cells were treated with MCE (a), brassicasterol (b), or HOEO (c) in the indicated concentrations for 3 h (lanes 3–6 or 3–7), followed by stimulation with LPS for 16 h (lanes 2–6 or 2–7). Band intensities relative to lane 1 in each blot were determined after normalization by the amount of actin and plotted as the respective histogram. (d) and (e), ELISA analysis for the concentration of NO (d) or PGE_2_ (e) in the culture medium. Cells were treated with 100 *μ*g/mL of MCE (Group 3) or 20 *μ*M of HOEO (Group 4) for 3 h, followed by stimulation with LPS (Groups 2, 3, and 4) for 16 h. Data represent the mean ± SD of three independent experiments. *P < 0.05 against Group 2. (f), the expression level of IL-1**β** mRNA analyzed by semiquantitative RT-PCR. Cells were treated as in (d) and (e). The expression of GAPDH was assayed simultaneously as the loading control. Fold represents the relative band intensity against lane 1 after normalization by the amount of GAPDH.

Inducible nitric oxide synthase (iNOS) and cyclooxygenase-2 (COX-2) are the major enzymes involved in the synthesis of the inflammatory mediators, NO, and PGE_2_, respectively, and are common markers of inflammation [12, 15, 16]. Thus, the dosage-dependent effects of MCE and brassicasterol on the inhibition of LPS-induced expression of iNOS and COX-2 in RAW 264.7 cells were characterized. These enzymes were highly expressed in LPS-stimulated cells (Figures 4(a) and 4(b), lane 2 versus lane 1). MCE obviously inhibited the LPS-elevated expression of iNOS in concentrations of 50–200 *μ*g/mL and that of COX-2 in concentrations of 100–200 *μ*g/mL (Figure 4(a), lanes 4–6 versus lane 2). Accordingly, 100 *μ*g/mL of MCE was considered as an effective dosage for the following experiments. In contrast, brassicasterol had no obvious inhibitory effect on the expression of iNOS and COX-2 at dosages of 10–100 *μ*M (Figure 4(b), lanes 3–6 versus lane 2). Thus, brassicasterol was not considered as a potential anti-inflammatory agent. In Figure 4(c), HOEO markedly suppressed LPS-induced expression of iNOS and COX-2 in concentrations of 5–30 *μ*M (lanes 4–7 versus lane 2). Thus, 20 *μ*M of HOEO was used for the subsequent experiments.

Whether MCE and HOEO could suppress LPS-stimulated NO and PGE_2_ secretion was also examined.  Figure 4(d) showed that LPS-induced NO secretion (Group 2) was significantly reduced by the treatment of MCE or HOEO (Groups 3 and 4). Similarly, MCE or HOEO obviously inhibited LPS-induced production of PGE_2_ in RAW 264.7 cells (Figure 4(e), Groups 3 and 4 versus Group 2). Moreover, both MCE and HOEO suppressed LPS-elevated expression of the mRNA of IL-1**β** (Figure 4(f), lanes 3 and 4 versus lane 2). These results further confirmed the anti-inflammatory activities of MCE and HOEO in LPS-stimulated macrophages.

**Figure 5:**
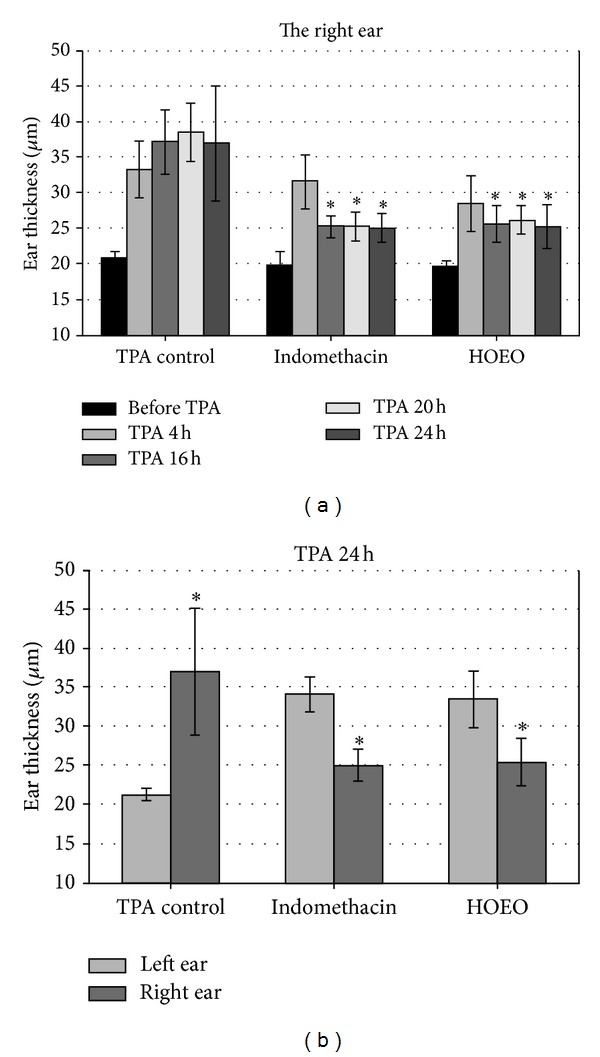
The anti-inflammatory effect of HOEO on TPA-induced ear edema of mice. Mice were treated with TPA on both ears for 1 h, followed by HOEO (300 *μ*g/ear) or indomethacin (500 *μ*g/ear) administration on the right ear, while the left ear was treated with the vehicle. In TPA control, TPA was applied to only the right ear, and both ears were treated with the vehicle at 1 h afterwards. Ear thickness was measured before and at 4 h, 16 h, 20 h, and 24 h after TPA treatment. Data represent the mean ± SD of each group (*N* = 6). (a) The comparison of the thickness of the right ear at different time points. *P < 0.05 against the same time point in the group of TPA control. (b) The comparison of the thickness of the right ear and the left ear at 24 h after TPA treatment. *P < 0.05 against the left ear of the same group.

Thus, the anti-inflammatory effect of HOEO was examined in animal models. As shown in Figure 5(a), the application of 300 *μ*g/ear of HOEO on the right ear significantly reduced the extent of mouse ear edema at 16 h, 20 h, and 24 h after TPA treatment and the effect was similar with that of 500 *μ*g/ear of indomethacin.  Figure 5(b) showed the comparison of the extents of edema between the right ear and the unmedicated left ear on the same group of mice at 24 h after TPA stimulation, and the findings also supported that HOEO effectively hindered TPA-induced edema. Consequently, these results confirmed the anti-inflammatory activity of HOEO *in vivo*.

### 3.3. Interference of MCE and HOEO with the Activation of the IKK/NF-*κ*B Pathway and MAPKs

The activation of nuclear factor-*κ*B (NF-*κ*B) plays a key role in mediating inflammatory responses. LPS is known to activate IKK (I*κ*B kinase), which in turn catalyzes the phosphorylation of I*κ*B (inhibitor of NF-*κ*B), leading to I*κ*B degradation and the release of NF-*κ*B, which enters the nucleus and activates the expression of pro-inflammatory factors such as iNOS, COX-2, and IL-1**β** [17–19].

**Figure 6:**
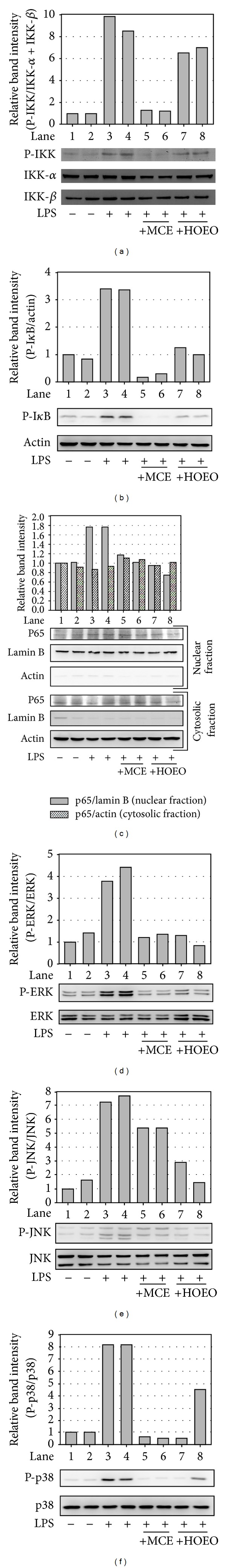
Inhibition of LPS-induced activation of the IKK/NF-*κ*B pathway and MAPKs by MCE or HOEO. Western blot was utilized to assay the expression of the indicated proteins in RAW 264.7 cells. Experiments were performed in duplicate. Cells were treated with 100 *μ*g/mL of MCE (lanes 5 and 6) or 20 *μ*M of HOEO (lanes 7 and 8) for 3 h, followed by stimulation with LPS (lanes 3–8) for 1 h or 2 h (c). (a) The analysis of phosphorylated IKK (P-IKK) and total **α** and **β** subunits of the IKK complex (IKK-**α** and IKK-**β**). (b) The analysis of phosphorylated I*κ*B (P-I*κ*B) and actin. (c) The analysis of p65, lamin B, and actin in the nuclear and cytosolic fractions of cells, respectively. (d), (e), and (f), the analysis of phosphorylated ERK (P-ERK) and total ERK (d), phosphorylated JNK (P-JNK) and total JNK (e), and phosphorylated p38 (P-p38) and total p38 (f), respectively. Band intensities relative to lane 1 in each blot were determined after normalization by the corresponding total protein, actin or lamin B as indicated in the respective histogram.

As revealed in Figures 6(a) and 6(b), MCE strongly inhibited LPS-induced phosphorylation of IKK (A) and I*κ*B (B) (lanes 5 and 6) as compared with cells treated with LPS alone (lanes 3 and 4). HOEO exhibited a less potent inhibitory effect on the activation of IKK (Figure 6(a), lanes 7 and 8 versus lanes 3 and 4), but still showed a substantial suppressive effect on the phosphorylation of I*κ*B (Figure 6(b), lanes 7 and 8 versus lanes 3 and 4). Therefore, LPS-induced I*κ*B phosphorylation was obviously inhibited by both MCE and HOEO. Furthermore, Figure 6(c) showed that the nuclear translocation of p65, an NF-*κ*B subunit, was apparently increased by LPS stimulation (lanes 3 and 4) as compared to untreated cells (lanes 1 and 2), whereas MCE (lanes 5 and 6) or HOEO (lanes 7 and 8) critically suppressed this LPS-induced effect, indicating that MCE and HOEO inhibited the shift of NF-*κ*B to the nucleus. Overall, these data suggest that MCE and HOEO inhibit the LPS-mediated activation of IKK/NF-*κ*B signaling.

Mitogen-activated protein kinases (MAPKs), including extracellular signal-regulated kinase (ERK)1/2, c-Jun N-terminal kinase (JNK), and p38, are also known to mediate the LPS-induced inflammatory responses [20–23]. Thus, whether MCE and HOEO could inhibit the activation of JNK, ERK, and p38 was examined. As shown in Figure 6(d), treatment of the cells with MCE (lanes 5 and 6) or HOEO (lanes 7 and 8) effectively inhibited the LPS-induced phosphorylation of ERK as compared with the LPS control (lanes 3 and 4). HOEO also markedly inhibited the phosphorylation of JNK (Figure 6(e), lanes 7 and 8 versus lanes 3 and 4), whereas MCE displayed a less strong inhibitory action on the activation of JNK (Figure 6(e), lanes 5 and 6 versus lanes 3 and 4).  Figure 6(f) manifested that both MCE (lanes 5 and 6) and HOEO (lanes 7 and 8) substantially suppressed the LPS-induced phosphorylation of p38 as compared with the control (lanes 3 and 4).

## 4. Discussion

This study scientifically proved that the crude extract of *F. polycarpa *is a potential anti-inflammatory agent. Animal tests confirmed the anti-inflammatory effect of MCE *in vivo*. Topical application of MCE for 12–20 h effectively ameliorated acute skin inflammation on mice ear. While compared with indomethacin, a medicine for inflammation, MCE showed a similar or even stronger effect in reducing ear edema of mice. Meanwhile, in RAW 264.7 macrophage line, MCE obviously inhibited LPS-stimulated expression or production of iNOS, COX-2, NO, PGE_2_, and IL-1**β**. Overall, these results support the traditional use of* F. polycarpa* as an herbal medicine in treating inflammatory-related diseases.

MCE obviously reduced the phosphorylation of IKK and I*κ*B and the nuclear translocation of NF-*κ*B. These findings suggested that MCE inhibited the IKK/NF-*κ*B pathway. MAPKs are another group of signaling pathways regulating inflammation. MCE critically suppressed LPS-induced activation of ERK and p38 and had a milder inhibitory effect on that of JNK. Generally, these results suggest that MCE interferes with the IKK/NF-*κ*B pathway and the MAPK pathways, which likely underlies, at least in part, the mechanism of its anti-inflammatory activity.

MCE was rich in triterpenoids as well as in phytosterols. It is rational to speculate that these components may contribute to the anti-inflammatory function of* F. polycarpa*. Thus, brassicasterol and a few major or feature triterpenes of MCE were characterized. Yet, brassicasterol did not exhibit an obvious anti-inflammatory activity, implying that brassicasterol may not contribute to the anti-inflammatory effect of MCE. Consistently, brassicasterol is known to be a phytosterol commonly present in many plants and foods [24–26], including those unknown to possess an anti-inflammatory activity. Thus, it is reasonable to infer that brassicasterol does not have an anti-inflammatory effect.

On the other hand, HOEO displayed a significant anti-inflammatory activity both *in vivo* and *in vitro*. HOEO obviously diminished TPA-induced ear edema in mice and inhibited the expression of proinflammatory markers in LPS-stimulated RAW 264.7 cells, including iNOS, COX-2, IL-1**β**, NO, and PGE_2_. Furthermore, in accordance with the action of MCE, HOEO interfered with the activation of the IKK/NF-*κ*B pathway and that of MAPKs ERK, JNK, and p38. Hence, HOEO likely represents one of the constituents contributing to the anti-inflammatory function of *F. polycarpa*. It is very likely that other components in MCE also play roles in the anti-inflammatory effect of* F. polycarpa* and deserve to be explored.

Interestingly, HODA, fatsicarpain D, and fatsicarpain F showed obvious inhibitory effects on the survival of RAW 264.7 cells, but they were found nontoxic to the normal liver line FL83B cell. The molecular mechanism for this effect is not clear. Nonetheless, promoting the death of macrophages without hurting other somatic cells could also be an effect of anti-inflammation. Thus, the actions, immunomodulatory effects, and molecular mechanisms of these molecules should be further investigated.

## 5. Conclusions

Evidence presented in this study suggests that the crude extract from the leaves and twigs of *Fatsia polycarpa* is a potent anti-inflammatory agent both* in vitro* and* in vivo*. The anti-inflammatory activity of MCE is probably not due to brassicasterol, a major phytosterol present in the extract, but is likely contributed by other components in the extract, such as triterpenoids like HOEO. The molecular mechanism underlying the effects of MCE and HOEO probably involves the inhibition of the IKK/NF-*κ*B pathway and the MAPKs. To further our understanding, more studies should be conducted to continuously explore the effective constituents in the extract of *F. polycarpa*.
